# Ultrasound-guided glenohumeral joint injections for shoulder pain in ALS: A case series

**DOI:** 10.3389/fneur.2022.1067418

**Published:** 2023-02-02

**Authors:** Katherine M. Burke, Amy S. Ellrodt, Benjamin C. Joslin, Pia P. Sanpitak, Claire MacAdam, Prabhav Deo, Kevin Ozment, Cristina Shea, Stephen A. Johnson, Doreen Ho, Samuel K. Chu, Ashwin N. Babu, Colin K. Franz, Sabrina Paganoni

**Affiliations:** ^1^Sean M. Healey & AMG Center for ALS at Massachusetts General Hospital, Department of Neurology, Harvard Medical School, Boston, MA, United States; ^2^Ken and Ruth Davee Department of Neurology, Northwestern University Feinberg School of Medicine, Chicago, IL, United States; ^3^Shirley Ryan AbilityLab, Chicago, IL, United States; ^4^Physical Medicine and Rehabilitation, Northwestern University Feinberg School of Medicine, Chicago, IL, United States; ^5^Department of Physical Medicine and Rehabilitation, Spaulding Rehabilitation Hospital, Boston, MA, United States; ^6^Massachusetts General Hospital Department of Orthopedics, Sports Medicine, Boston, MA, United States; ^7^VA Boston Healthcare System, Boston, MA, United States

**Keywords:** amyotrophic lateral sclerosis, motor neuron disease, shoulder pain, frozen shoulder, adhesive capsulitis

## Abstract

**Introduction:**

Shoulder pain is a common secondary impairment for people living with ALS (PALS). Decreased range of motion (ROM) from weakness can lead to shoulder pathology, which can result in debilitating pain. Shoulder pain may limit PALS from participating in activities of daily living and may have a negative impact on their quality of life. This case series explores the efficacy of glenohumeral joint injections for the management of shoulder pain due to adhesive capsulitis in PALS.

**Methods:**

People living with ALS and shoulder pain were referred to sports medicine-certified physiatrists for diagnostic evaluation and management. They completed the Revised ALS Functional Rating Scale and a questionnaire asking about their pain levels and how it impacts sleep, function, and quality of life at baseline pre-injection, 1-week post-injection, 1 month post-injection, and 3 months post-injection.

**Results:**

We present five cases of PALS who were diagnosed with adhesive capsulitis and underwent glenohumeral joint injections. Though only one PALS reported complete symptom resolution, all had at least partial symptomatic improvement during the observation period. No complications were observed.

**Conclusions:**

People living with ALS require a comprehensive plan to manage shoulder pain. Glenohumeral joint injections are safe and effective for adhesive capsulitis in PALS, but alone may not completely resolve shoulder pain. Additional therapies to improve ROM and reduce pain should be considered.

## 1. Introduction

Amyotrophic lateral sclerosis (ALS) is a fatal neurodegenerative disease (1). The management of ALS is largely focused on symptom management as current FDA-approved treatments to slow disease progression have modest efficacy (1).

Amyotrophic lateral sclerosis symptoms include muscle weakness and atrophy, reduced range of motion, spasticity, and cramping leading to progressive disability. Pain is a common secondary complication of ALS from musculoskeletal dysfunction due to limited mobility, loss of range of motion, and difficulty with positioning in bed, chairs, or wheelchairs (2). Progressive, often non-uniform, weakness likely also contributes by disrupting agonist-antagonist muscle pair equilibrium leading to increased stress on the joint and surrounding structures. Pain negatively affects the quality of life for both PALS and their caregivers (2–7) The most commonly reported loci of pain in PALS are the lower back, neck, legs, and shoulders (2, 8) Shoulder pain is reported by ~20% of the total ALS population (8). Many PALS develop shoulder pain due to weakness of the periscapular muscles with subsequent loss of range of motion, subluxation, and development of shoulder pathology, including adhesive capsulitis or “frozen shoulder.”

Adhesive capsulitis is a complex disorder that involves pain and restricted range of motion (ROM) of the shoulder (9–11) and can lead to considerable disability. The capsule surrounding the glenohumeral (GH) joint stiffens and becomes inflamed, which results in a decreased range of motion (ROM) at the shoulder. There are overlapping stages of adhesive capsulitis. In the earlier stages, patients present with severely restricted ROM and significant pain, often in the absence of injury. In the later stages, restrictions in ROM remain, but with improvements in pain. As ROM and pain improve, there may be continued evidence of a tight joint space, but no evidence of synovitis (10). Adhesive capsulitis is largely a clinical diagnosis, with any imaging done to exclude other potential diagnoses (9).

For individuals without ALS, the management of adhesive capsulitis involves exercises and supervised physical therapy focused on gentle ROM and periscapular stabilization. First-line medications typically prescribed to treat pain in ALS are non-steroidal anti-inflammatory agents (NSAIDs) and non-opioid analgesics (4, 12). Early corticosteroid injection to the GH joint should be considered for moderate to severe symptoms (13, 14), as there is evidence suggesting improved outcomes with earlier steroid injection (15). This in-clinic procedure is fast and safe. It is routinely performed in the seated position, which is preferable for PALS who may be in a power wheelchair and/or have limited tolerance for the supine position. Adverse effects such as bleeding or infection are rare when performed with proper clean technique. In cases where conservative measures are ineffective, surgical interventions such as arthroscopic release and manipulation under anesthesia are considered, especially in the early stages when the pain can be debilitating (16, 17).

To the best of our knowledge, there is no evidence from controlled trials that would substantiate a standard best practice for addressing shoulder pain in people with ALS. Thus, the management of shoulder pain has long been based on the individual clinician's experience and preferences with very little empirically-derived evidence to support the implementation of one therapeutic method over another. When PALS present with progressive shoulder pain, accompanying restrictions in ROM, and no history of trauma, referral to physiatry can help to assess the pain generator (e.g., adhesive capsulitis vs. other shoulder pathology) and help guide the intervention. There is growing evidence to support the use of GH steroid injections for adhesive capsulitis for those without ALS (13–17).

The purpose of this study was to assess the efficacy of ultrasound-guided GH joint injections for treating shoulder pain due to adhesive capsulitis in PALS. We present a case series of five patients referred for physiatric evaluation and consideration of GH joint injections as part of their clinical care. Participants completed surveys pre- and post-injections regarding their pain levels and impact on function.

## 2. Methods

Participants were recruited from the Sean M. Healey & AMG Center for ALS at Massachusetts General Hospital and the Northwestern University Feinberg School of Medicine ALS Clinic. Eligible participants were informed of the study and study staff consented interested participants. Both the Mass General Brigham Institutional Review Board (IRB) and the Northwestern IRB approved this study.

Participants were asked to participate in this study if they had ALS and a clinical suspicion of adhesive capsulitis (pain with restricted ROM, without a history of trauma). Participants were seen by physiatrists certified in Sports Medicine by the American Board of Physical Medicine and Rehabilitation for diagnostic confirmation and consideration of GH steroid injections. The physiatrists completed a history, physical, and ultrasound examinations to assess the likelihood of adhesive capsulitis vs. other common shoulder pathologies (such as rotator cuff or biceps tendinopathy, periscapular myofascial pain, etc.). Once a clinical diagnosis of adhesive capsulitis was established, an ultrasound-guided GH joint injection was performed. For Cases 1–3, a solution of 4 ml of 1% Lidocaine and 1 ml (40 mg) of Kenalog was injected into the affected joints. For Cases 4 and 5, a solution of 2 ml of 1% Lidocaine, 2 ml of 0.25% Bupivacaine, and 1 ml (40 mg) of Kenalog was injected into the involved joints. Differences were due to the preference of the physiatrist performing the intervention. The study duration was from May 2019 to August 2021. Enrollment was limited by the COVID-19 pandemic, as sites had temporary holds on the observational studies, and patient care shifted to telemedicine visits.

Participants were asked to complete the revised ALS Functional Rating Scale (ALSFRS-R) and a questionnaire that assessed pain, sleep, function, and quality of life (Supplementary material) at baseline before undergoing the GH joint injections. They were then asked to complete the same questionnaires at 1-week post-injection and 1-month post-injection. There was an optional phone call at 3 months to complete the same questionnaires.

Electronic medical record review was available for all participants, from which information regarding their ALS diagnosis, disease duration, and current functional status was gathered.

## 3. Results

Of nine participants enrolled in the study, three initially agreed but ultimately declined the referral for GH joint injections and were provided with ROM and stretching exercises by the clinic's physical therapists. Follow-up of these individuals was not pursued as part of this study. Another participant was seen by physiatry: diagnostic evaluation pointed to myofascial pain with muscle spasms as the most likely pain generator. For this reason, the participant underwent bilateral trapezius, infraspinatus, and levator scapulae trigger point injections rather than GH joint injections. This participant did have resolution of his shoulder pain 18 days post-injection, but this was not sustained 2 months post-injection. We report on the remaining five participants who were diagnosed by sports medicine-certified physiatrists with adhesive capsulitis and did undergo ultrasound-guided GH joint steroid injections. None of the participants experienced procedure-related complications. Average pain scores and impact on sleep and quality of life for each participant are shown in Table 1. Complete data for each participant are shown in Supplementary Tables 1–5.

**Table 1:** Pain and impact on sleep, function, and quality of life.

	**Case 1**	**Case 2**	**Case 3**	**Case 4**	**Case 5**
**Average pain 0–10 (R/L)**					
Pre-injection (days from baseline)	7/7 (−19)	7/0 (−22)	3/4 (−3)	6/5 (−18)	1/7 (0)
1st follow-up (days from baseline)	4/7 (7)	2/0 (7)	0/0 (7)	1/2 (10)	0/0 (11)
2nd follow-up (days from baseline)	5/7 (28)	2/0 (21)	2/3 (25)	4/4 (31)	0/0 (28)
3rd follow-up (days from baseline)	0/0 (74)	Not collected	0/0 (87)	6/6 (87)	0/0 (87)
**Max pain 0**–**10 (R/L)**					
Pre-injection	9/9	9/0	4/5	8/8	1/8
1st follow-up	8/7	4/0	5/5	4/4	0/0
2nd follow-up	8/8	5/0	2/3	8/8	0/0
3rd follow-up	5/5	Not collected	10/10	8/6	0/0
**My shoulder pain keeps me from getting enough sleep at night**					
Pre-injection	Often	Sometimes	Rarely	Always	Always
1st follow-up	Never	Rarely	Rarely	Never	Never
2nd follow-up	Never	Sometimes	Never	Never	Never
3rd follow-up	Never	Not collected	Never	Never	Never
**My shoulder pain limits my ability to complete daily hygiene**					
Pre-injection	Always	Often	Always	Always	Never
1st follow-up	Often	Rarely	Always	Never	Never
2nd follow-up	Sometimes	Sometimes	Never	Never	Never
3rd follow-up	Rarely	Not collected	Always	Never	Never
**My shoulder pain limits my ability to get dressed each day**					
Pre-injection	Always	Often	Always	Always	Always
1st follow-up	Often	Sometimes	Always	Rarely	Never
2nd follow-up	Sometimes	Sometimes	Never	Rarely	Never
3rd follow-up	Always	Not collected	Always	Sometimes	Never
**My shoulder pain limits me from leaving the house for other activities**					
Pre-injection	Always	Rarely	Often	Sometimes	Never
1st follow-up	Never	Rarely	Always	Rarely	Never
2nd follow-up	Never	Sometimes	Never	Rarely	Never
3rd follow-up	Never	not collected	Rarely	Rarely	Never
**My shoulder pain has a negative impact on my quality of life**					
Pre-injection	Always	Often	Always	Always	Never
1st follow-up	Often	Sometimes	Sometimes	Never	Never
2nd follow-up	Often	Sometimes	Never	Never	Never
3rd follow-up	Always	not collected	Always	Never	Never

### 3.1. Case 1

The participant was a 41-year-old woman with upper motor neuron predominant limb onset sporadic ALS, meeting El Escorial criteria for definite ALS. She used a wheelchair for mobility and was dependent on caregivers for activities of daily living (ADLs). ALS symptom onset was over 3 years prior with left-hand weakness and clawing. She had bilateral upper and lower extremity weakness with atrophy (left greater than right), and spasticity throughout her extremities. She presented with atraumatic bilateral shoulder pain, right greater than left, with passive range of motion restrictions. She was taking NSAIDs as needed and undergoing physical therapy with shoulder range of motion and stretching exercises. Physiatry evaluation confirmed the diagnosis of adhesive capsulitis, and she underwent a right GH joint injection with ultrasound guidance. Post-injection, she demonstrated improvements in shoulder pain, which were sustained at the 3-month visit, as well as sustained improvements in sleep, function, and quality of life (Supplementary Table 1).

### 3.2. Case 2

The participant was a 42-year-old man with limb onset sporadic ALS, meeting El Escorial criteria for probable ALS—laboratory supported. He was ambulatory with a single-point cane and had modified independence with ADLs. ALS onset was over 2 years prior with lower extremity weakness. He presented with upper and lower extremity weakness and atrophy, with brisk reflexes in bilateral lower extremities. He reported atraumatic right shoulder pain that started a few months before, with limited ROM. He was not taking any medications and was not participating in physical therapy. He had been prescribed shoulder range of motion and stretching exercises at a multidisciplinary clinic but was unable to perform them due to pain. Physiatry evaluation confirmed the diagnosis of adhesive capsulitis and he underwent a right GH joint injection with ultrasound guidance. Post-injection, he demonstrated improvements in shoulder pain, which were sustained at the 1-month visit, as well as improvements in sleep, function, and quality of life (Supplementary Table 2). He initiated a stretching program for bilateral shoulders post-injection.

### 3.3. Case 3

The participant was a 57-year-old man with limb onset sporadic ALS, meeting El Escorial criteria for probable ALS—laboratory supported. ALS symptom onset was over 3 years prior with lower extremity weakness. He was ambulatory without an assistive device and required assistance with ADLs due to bilateral upper extremity weakness. He had bilateral upper and lower extremity weakness, with increased reflexes in bilateral lower and left upper extremities. He presented with bilateral atraumatic shoulder pain, left greater than right. He had been prescribed shoulder range of motion and stretching exercises at a multidisciplinary clinic but was unable to perform them due to pain. He was not taking any pain medications. Physiatry evaluation confirmed the diagnosis of adhesive capsulitis and he underwent bilateral GH joint injections with ultrasound guidance. Overall, the pain was improved in bilateral shoulders post-injection, and this was sustained at the 3-month visit. At 1 month, the pain was not impacting sleep, function, and quality of life, but this was not sustained at 3 months (Supplementary Table 3). He was stretching at the 1-month mark, but this was not sustained at 3 months.

### 3.4. Case 4

The participant was a 49-year-old man with upper motor neuron predominant limb onset sporadic ALS, meeting El Escorial criteria for probable ALS—laboratory supported. He presented with mild bilateral upper extremity weakness without muscle atrophy, as well as diffuse muscle spasticity characterized by a slight catch at the end range of the passive range of motion. He was power wheelchair dependent for community mobility and used either a cane or a power wheelchair for household mobility. He had modified independence with ADLs. He presented with 6 months of progressively worsening atraumatic bilateral anterolateral shoulder pain and stiffness, left greater than right. He had passive and active ROM restrictions bilaterally. He had been taking ibuprofen as needed for several years. He had been prescribed shoulder range of motion and stretching exercises at a multidisciplinary clinic but was not able to comply with them due to pain. He underwent bilateral GH joint injections with ultrasound guidance. He exhibited improvements in the impact of shoulder pain that lasted only 1 month, but the improvements in his quality of life and function were largely sustained at the 3-month follow-up (Supplementary Table 4).

### 3.5. Case 5

The participant is a 58-year-old woman with upper motor neuron predominant limb onset ALS, meeting El Escorial criteria for definite ALS. Despite having no family history of ALS or dementia, she was later found to carry a hexanucleotide repeat expansion in the C9orf72 gene (>145 repeats). She had some hand intrinsic muscle atrophy and moderate bilateral arm weakness (right worse than left). She had diffusely increased muscle tone treated with oral baclofen. She was ambulatory and required assistance with ADLs due to upper extremity weakness. She presented with atraumatic left shoulder pain and stiffness for 2 months. She managed her pain with a nightly dose of ibuprofen and had not yet started physical therapy before the initial referral for injection. She had limited active and passive shoulder ROM. She underwent left GH joint injections with ultrasound guidance. In early and late follow-ups, the patient reported the resolution of shoulder pain and it was no longer impacting sleep. This allowed her to engage in a shoulder home exercise program provided by ALS interdisciplinary clinic occupational therapist with assistance from her family. Resolution of pain and impact on sleep, function, and quality of life were sustained at the 3-month visit (Supplementary Table 5).

## 4. Discussion

In this case series, we present empiric evidence of the benefit of GH joint steroid injections for PALS who present with shoulder pain in the setting of adhesive capsulitis. All study participants presented here reported improvement in their pain levels and a positive impact on sleep, function, or quality of life. One of the participants (Case 5) reported a complete resolution of her shoulder pain. There were no adverse events reported by the participants in this study. These results demonstrate the safety of, and suggest a role for, ultrasound-guided GH joint steroid injections for PALS with adhesive capsulitis, though additional measures may be needed for further pain management.

The development of shoulder pain from adhesive capsulitis in PALS is likely a secondary effect of their disease resulting from multiple factors such as decreased shoulder muscle strength, muscle atrophy, and reduced activity levels. The progressive lack of motion may contribute to the inflammation and tightening of the joint capsule. Similar pathology has been reported in both stroke and Parkinson's disease where there is motor dysfunction involving the shoulder (18, 19). Motion is critical to maintain and restore ROM (13, 16). Early in the disease course, PALS meet with physical therapists and occupational therapists in the multidisciplinary clinic to review the importance of a regular range of motion exercise program for all joints, including the shoulders. For individuals with upper extremity weakness, especially the shoulder, the range of motion exercises become even more crucial, and the physical therapists or occupational therapists will review the range of motion and stretching program with patients and train their caregivers if they are not able to complete the exercises on their own. Unfortunately, some PALS still present with shoulder pain. For these PALS, stretching and range of motion exercises are reinforced, and strategies to increase compliance can be helpful (20). Shoulder approximation sleeves can also be used to help support the shoulder and manage pain, as well as modalities such as transcutaneous electrical nerve stimulation (TENS) devices. However, while these tools and strategies have shown promise, these interventions are not always enough to completely relieve pain or improve function (20). Unfortunately, there may be limited ability to complete the recommended exercises due to disease progression or pain. Often, the shoulder will become quite painful, will limit the ability to participate in ADLs, including daily hygiene, and may impact sleep quality and overall quality of life. When pain has become a limiting factor, the provider is challenged to make appropriate recommendations for adequate pain control and to optimize the person's ability to participate in ADLs. This study highlights the need for a multifaceted approach, which may include GH joint injections, as well as education on the cause of shoulder pain and the importance of a long-term stretching program to maintain a functional ROM to minimize risk for re-freezing or ongoing pain.

Referral to musculoskeletal specialists for the consideration for steroid injections should be considered for PALS with shoulder pain. The procedure offers the advantages of being a fast, safe, in-office procedure that requires no ionizing radiation or sedation. Evidence in non-ALS populations shows improved pain relief with steroid injections compared to manual therapy and exercise without injection (21). Shoulder injections have also been shown to be beneficial for hemiplegic shoulder pain in patients status post-stroke (22). With pain relief from the injections, the person may be able to tolerate ROM and stretching exercises, which will restore motion and in turn may further improve pain, and help to restore function and quality of life. Appropriate pain relief with localized steroid injections also offers a strategy to reduce reliance on systemic pain medications with potential GI, renal, and neurologic side effects.

It is important to minimize the delays in referrals to musculoskeletal specialists, given the emerging evidence of improved outcomes with earlier steroid injections in people without ALS, as well as improved efficacy compared to manual therapy with exercise (14, 21). While early diagnosis and intervention is ideal, our cohort suggests that GH joint injection for adhesive capsulitis likely benefits PALS across the progression of ALS. If employed in early ALS, GH injection may allow a person to maintain ROM, functional independence, and participation in active physical therapy programs. In later-stage ALS, GH injection may offer improved passive ROM and caregiver ability to assist with ADLs, such as dressing and bathing. Throughout all ALS stages, the pain-reducing effect of GH injection may have benefits on activity, sleep, and mood.

Our study was limited by the small number of participants and case series design. The ongoing COVID-19 pandemic also limited participation, such that we were not able to collect the range of motion measurements as the participants completed their post-injection visits over the telephone, and many did not present to the clinic again during the study period due to COVID-19 restrictions. Further research is also needed to compare the outcomes between those who choose not to have the injections to those who do choose this intervention. We did not collect this information as part of this study. Finally, as more evidence emerges regarding specific injection techniques and steroid dose for people without ALS, it will be important to explore these same questions in the ALS population.

This case series supports the safety and efficacy of GH joint injections for adhesive capsulitis in PALS. Larger studies to determine the prevalence of adhesive capsulitis vs. other pathologies and to optimize comprehensive management plans aimed at complete shoulder pain resolution are needed.

## Data availability statement

The original contributions presented in the study are included in the article/Supplementary material, further inquiries can be directed to the corresponding author.

## Author contributions

KB, BJ, and CF: concept and design, data collection, data analysis, drafting of the manuscript, and critical review of the manuscript. AE, AB, and SP: concept and design, data collection, data analysis, and critical review of the manuscript. PS, PD, KO, and SC: data collection, data analysis, and critical review of the manuscript. CM, CS, SJ, and DH: data analysis and critical review of the manuscript. All authors contributed to the article and approved the submitted version.
