# Supplementary material for: Ultrasound-guided glenohumeral joint injections for shoulder pain in ALS: A case series
Source: Front Neurol. 2023 Feb 2;13:1067418. doi: 10.3389/fneur.2022.1067418 (PMC9940635; doi:10.3389/fneur.2022.1067418)
Supplement: Supplementary file 1 [file Data_Sheet_1.pdf]

Supplementary Material

1 Supplementary Tables

Supplementary Table 1. Case 1.

	Pre-injection*	1st follow-up post injection	2nd follow-up post injection	3rd follow-up post injection
Time from Baseline (days)	-19	7	28	74
Pain 0-10 (R/L)				
Average throughout day	7/7	4/7	5/7	0/0
Maximum throughout day	9/9	8/7	8/8	5/5
Lying down at rest	3/3	0/0	0/0	0/0
Sitting at rest	3/3	0/0	0/0	0/0
Eating	8/8	4/0	4/0	0/0
Getting dressed	10/10	8/7	1/1	5/5
During transfers	10/10	8/7	8/7	0/0
Riding in car	8/8	0/0	0/0	0/0
Walking	NA	NA	NA	NA
ALSFRS-R fine and gross motor questions				
4. Handwriting	2	1	1	0
5. Cutting and handling utensils	1	0	0	1
6. Dressing and hygiene	0	1	1	1
7. Turning in bed and adjusting bed clothes	1	1	1	1
8. Walking	1	1	1	1
9. Climbing stairs	0	0	0	0
Total (0-24)	5	4	4	4
My shoulder pain keeps me from getting enough sleep at night.	Often	Never	Never	Never
My shoulder pain limits my ability to complete daily hygiene.	Always	Often	Sometimes	Rarely
My shoulder pain limits my ability to get dressed each day.	Always	Often	Sometimes	Always
My shoulder pain limits me from leaving the house for other activities.	Always	Never	Never	Never
My shoulder pain has a negative impact on my quality of life.	Always	Often	Often	Always

*Did not complete survey on day of baseline, but patient reported right shoulder pain was 10/10 at its worst on day of injections.

Supplementary Table 2. Case 2.

	Pre-injection*	1st follow-up post injection	2nd follow-up post injection
Time (days)	-22	7	21
Pain 0-10 (R/L)			
Average throughout day	7/0	2/0	2/0
Maximum throughout day	9/0	4/0	5/0
Lying down at rest	3/0	1/0	2/0
Sitting at rest	3/0	1/0	2/0
Eating	3/0	2/0	2/0
Getting dressed	6/0	3/0	4/0
During transfers	7/0	3/0	4/0
Riding in car	4/0	2/0	2/0
Walking	5/0	2/0	2/0
ALSFRS-R fine and gross motor questions			
4. Handwriting	0	0	0
5. Cutting and handling utensils	2	2	2
6. Dressing and hygiene	2	2	2
7. Turning in bed and adjusting bed clothes	3	3	3
8. Walking	2	2	2
9. Climbing stairs	1	1	1
Total (0-24)	10	10	10
My shoulder pain keeps me from getting enough sleep at night.	Sometimes	Rarely	Sometimes
My shoulder pain limits my ability to complete daily hygiene.	Often	Rarely	Sometimes
My shoulder pain limits my ability to get dressed each day.	Often	Sometimes	Sometimes
My shoulder pain limits me from leaving the house for other activities.	Rarely	Rarely	Sometimes
My shoulder pain has a negative impact on my quality of life.	Often	Sometimes	Sometimes

*Did not complete survey on day of baseline, but patient reported pain 2/10 at baseline visit with a maximum pain of 5/10 in the right shoulder.

Supplementary Table 3. Case 3.

	Pre-injection*	1st follow-up post injection	2nd follow-up post injection	3rd follow-up post injection
Time (days)	-3	7	25	87
Pain 0-10 (R/L)				
Average throughout day	3/4	0/0	2/3	0/0
Maximum throughout day	4/5	5/5	2/3	10/10
Lying down at rest	3/5	0/0	0/0	0/0
Sitting at rest	2/4	0/0	0/0	0/0
Eating	3/5	5/5	0/0	1/1
Getting dressed	7/8	5/5	2/3	4/4
During transfers	5/7	0/0	2/3	0/0
Riding in car	3/4	0/0	0/0	3/3
Walking	2/4	0/0	0/0	3/3
ALSFRS-R fine and gross motor questions				
4. Handwriting	3	3	3	1
5. Cutting and handling utensils	1	1	1	1
6. Dressing and hygiene	1	0	1	1
7. Turning in bed and adjusting bed clothes	2	1	1	1
8. Walking	3	3	3	3
9. Climbing stairs	1	0	1	1
Total (0-24)	11	8	10	8
My shoulder pain keeps me from getting enough sleep at night.	Rarely	Rarely	Never	Never
My shoulder pain limits my ability to complete daily hygiene.	Always	Always	Never	Always
My shoulder pain limits my ability to get dressed each day.	Always	Always	Never	Always
My shoulder pain limits me from leaving the house for other activities.	Often	Always	Never	Rarely
My shoulder pain has a negative impact on my quality of life.	Always	Sometimes	Never	Always

*Did not complete survey on day of baseline visit as pre-injection outcomes were collected just three days prior. He did report that his pain levels were unchanged.

Supplementary Table 4. Case 4.

	Pre-injection*	Baseline Day of Injections	1st follow-up post injection	2nd follow-up post injection	3rd follow-up post injection
Time (days)	-18		10	31	87
Pain 0-10 (R/L)					
Average throughout day	6/5	2/5	1/2	4/4	6/6
Maximum throughout day	8/8	5/5	4/4	8/8	8/6
Lying down at rest	NA/5	0/0	0/0	0/0	0/0
Sitting at rest	5/5	2/2	0/0	0/0	0/0
Eating	NA/5	0/0	0/0	0/0	0/0
Getting dressed	5/8	3/3	1/2	2/2	4/2
During transfers	5/8	0/0	0/0	0/0	0/0
Riding in car	NA/5	2/2	0/0	0/0	0/0
Walking	2/5	5/5	3/5	8/8	0/0
ALSFRS-R fine and gross motor questions					
4. Handwriting	4		4	4	4
5. Cutting and handling utensils	4		4	4	4
6. Dressing and hygiene	2		2	2	2
7. Turning in bed and adjusting bed clothes	3		3	3	3
8. Walking	2		2	2	2
9. Climbing stairs	1		1	1	1
Total (0-24)	16		16	16	16
My shoulder pain keeps me from getting enough sleep at night.	Always	Never	Never	Never	Never
My shoulder pain limits my ability to complete daily hygiene.	Always	Always	Never	Never	Never
My shoulder pain limits my ability to get dressed each day.	Always	Sometimes	Rarely	Rarely	Sometimes
My shoulder pain limits me from leaving the house for other activities.	Sometimes	Often	Rarely	Rarely	Rarely
My shoulder pain has a negative impact on my quality of life.	Always	Sometimes	Never	Never	Never

*Screening and Baseline were completed on different days and patient completed survey on pain and function at both visits. ALSFRS-R was not repeated at the baseline visit.

Supplementary Table 5. Case 5.

	Pre-injection*	1st follow-up post injection**	2nd follow-up post injection	3rd follow-up post injection
Time (days)	0	11	28	87
Pain 0-10 (R/L)				
Average throughout day	1/7	0/0	0/0	0/0
Maximum throughout day	1/8	0/0	0/0	0/0
Lying down at rest	0/7	0/0	0/0	0/0
Sitting at rest	0/0	0/0	0/0	0/0
Eating	0/0	0/0	0/0	0/0
Getting dressed	0/2	0/0	0/0	0/0
During transfers	0/0	0/0	0/0	0/0
Riding in car	0/0	0/0	0/0	0/0
Walking	0/0	0/0	0/0	0/0
ALSFRS-R fine and gross motor questions				
4. Handwriting	0	0	0	0
5. Cutting and handling utensils	0	0	0	0
6. Dressing and hygiene	0	0	0	0
7. Turning in bed and adjusting bed clothes	2	2	2	0
8. Walking	3	3	2	1
9. Climbing stairs	0	0	0	0
Total (0-24)	5	5	4	1
My shoulder pain keeps me from getting enough sleep at night.	Always	Never	Never	Never
My shoulder pain limits my ability to complete daily hygiene.	Never	Never	Never	Never
My shoulder pain limits my ability to get dressed each day.	Always	Never	Never	Never
My shoulder pain limits me from leaving the house for other activities.	Never	Never	Never	Never
My shoulder pain has a negative impact on my quality of life.	Never	Never	Never	Never

*Patient completed pre-injection outcomes on same day as injection (baseline).

**Patient Reported No Pain starting 1st follow-up post injection.

2 Supplementary Data - Survey

Shoulder Survey

A. Do you have pain in your **RIGHT** shoulder? Yes No

If no, please skip to section B.

What is your average pain level in your **right** shoulder throughout the day?

0 1 2 3 4 5 6 7 8 9 10

What is the maximum pain level you experience in your **right** shoulder throughout the day?

0 1 2 3 4 5 6 7 8 9 10

On a scale of 0-10 with 0 being no pain and 10 being the worst pain imaginable, please rate your ***average RIGHT*** shoulder pain during the following activities:

Lying down at rest	N/A 0 1 2 3 4 5 6 7 8 9 10
Sitting at rest	N/A 0 1 2 3 4 5 6 7 8 9 10
Eating	N/A 0 1 2 3 4 5 6 7 8 9 10
Getting dressed	N/A 0 1 2 3 4 5 6 7 8 9 10
During transfers	N/A 0 1 2 3 4 5 6 7 8 9 10
Riding in a car	N/A 0 1 2 3 4 5 6 7 8 9 10
Walking	N/A 0 1 2 3 4 5 6 7 8 9 10

B. Do you have pain in your **LEFT** shoulder? Yes No

If no, please skip to section C.

What is your average pain level in your **left** shoulder throughout the day?

0 1 2 3 4 5 6 7 8 9 10

What is the maximum pain level you experience in your **left** shoulder throughout the day?

0 1 2 3 4 5 6 7 8 9 10

On a scale of 0-10 with 0 being no pain and 10 being the worst pain imaginable, please rate your ***average LEFT*** shoulder pain during the following activities:

Lying down at rest	N/A 0 1 2 3 4 5 6 7 8 9 10
Sitting at rest	N/A 0 1 2 3 4 5 6 7 8 9 10
Eating	N/A 0 1 2 3 4 5 6 7 8 9 10
Getting dressed	N/A 0 1 2 3 4 5 6 7 8 9 10
During transfers	N/A 0 1 2 3 4 5 6 7 8 9 10
Riding in a car	N/A 0 1 2 3 4 5 6 7 8 9 10
Walking	N/A 0 1 2 3 4 5 6 7 8 9 10

C. Please rate the following:

My shoulder pain keeps me from getting enough sleep at night.	Never	Rarely	Sometimes	Often	Always
My shoulder pain limits my ability to complete daily hygiene.	Never	Rarely	Sometimes	Often	Always
My shoulder pain limits my ability to get dressed each day.	Never	Rarely	Sometimes	Often	Always

My shoulder pain limits me from leaving the house for other activities.	Never	Rarely	Sometimes	Often	Always
My shoulder pain has a negative impact on my quality of life.	Never	Rarely	Sometimes	Often	Always

D. Please answer the following questions:

Do you do stretches or range of motion exercises for your shoulders? Yes No

If yes, how often?

a. Never
b. Occasionally (once a month to once a week)
c. Often (2-5 days a week)
d. Daily

If no, what are the barriers to doing stretches or range of motion exercises? (*check all that apply*)

- ☐ The exercises cause pain
- ☐ I don't have time
- ☐ I don't know how to do the exercises
- ☐ I cannot do the exercises by myself and I don't have people to help me
- ☐ I am not interested in exercising
- ☐ Other ____

Did you receive information about the importance of shoulder stretching or range of motion exercises for people with ALS? Yes No

If yes, where? (*check all that apply*)

- ☐ ALS Clinic or other healthcare setting (doctor, nurse, PT)
- ☐ Online
- ☐ Other ____
